# PKCα Modulates Epithelial-to-Mesenchymal Transition and Invasiveness of Breast Cancer Cells Through ZEB1

**DOI:** 10.3389/fonc.2019.01323

**Published:** 2019-11-27

**Authors:** María Candelaria Llorens, Fabiana Alejandra Rossi, Iris Alejandra García, Mariana Cooke, Martin C. Abba, Cynthia Lopez-Haber, Laura Barrio-Real, María Victoria Vaglienti, Mario Rossi, José Luis Bocco, Marcelo G. Kazanietz, Gastón Soria

**Affiliations:** ^1^Centro de Investigaciones en Bioquímica Clínica e Inmunología, CIBICI-CONICET, Córdoba, Argentina; ^2^Departamento de Bioquímica Clínica, Facultad de Ciencias Químicas, Universidad Nacional de Córdoba, Córdoba, Argentina; ^3^Instituto de Investigación en Biomedicina de Buenos Aires, IBioBA-CONICET, Partner Institute of the Max Planck Society, Buenos Aires, Argentina; ^4^Translational Medicine Research Institute (IIMT), CONICET, Facultad de Ciencias Biomédicas, Universidad Austral, Buenos Aires, Argentina; ^5^Department of Systems Pharmacology and Translational Therapeutics, Perelman School of Medicine, University of Pennsylvania, Philadelphia, PA, United States; ^6^Centro de Investigaciones Inmunológicas Básicas y Aplicadas, CONICET, Universidad Nacional de La Plata, La Plata, Argentina

**Keywords:** epithelial-to-mesenchymal transition, EMT, metastasis, breast cancer, triple negative, TNBC, PKCα, ZEB1

## Abstract

ZEB1 is a master regulator of the Epithelial-to-Mesenchymal Transition (EMT) program. While extensive evidence confirmed the importance of ZEB1 as an EMT transcription factor that promotes tumor invasiveness and metastasis, little is known about its regulation. In this work, we screened for potential regulatory links between ZEB1 and multiple cellular kinases. Exploratory *in silico* analysis aided by phospho-substrate antibodies and ZEB1 deletion mutants led us to identify several potential phospho-sites for the family of PKC kinases in the N-terminus of ZEB1. The analysis of breast cancer cell lines panels with different degrees of aggressiveness, together with the evaluation of a battery of kinase inhibitors, allowed us to expose a robust correlation between ZEB1 and PKCα both at mRNA and protein levels. Subsequent validation experiments using siRNAs against PKCα revealed that its knockdown leads to a concomitant decrease in ZEB1 levels, while ZEB1 knockdown had no impact on PKCα levels. Remarkably, PKCα-mediated downregulation of ZEB1 recapitulates the inhibition of mesenchymal phenotypes, including inhibition in cell migration and invasiveness. These findings were extended to an *in vivo* model, by demonstrating that the stable knockdown of PKCα using lentiviral shRNAs markedly impaired the metastatic potential of MDA-MB-231 breast cancer cells. Taken together, our findings unveil an unforeseen regulatory pathway comprising PKCα and ZEB1 that promotes the activation of the EMT in breast cancer cells.

## Introduction

Epithelial-to-mesenchymal transition (EMT) is an essential program of normal embryonic development, tissue regeneration, organ fibrosis and wound healing (1, 2). Activation of the EMT program is also a critical step during metastatic expansion and for the generation of tumor cells with stem cell properties that play a major role in resistance to cancer treatment (3–7). EMT is a highly dynamic process by which epithelial cells undergo molecular changes that promote the acquisition of a mesenchymal phenotype characterized by the disruption of cell–cell adhesion, loss of cellular polarity, remodeling of the cytoskeleton, and changes in cell–matrix adhesion, together with enhanced migratory and invasive properties (1, 3–5, 8). Several master regulatory programs have been discovered to play key roles in cancer progression and EMT, which can be activated by diverse signals, including TGF-β, Wnt, and TKR (tyrosine kinase receptor) ligands. These external signals regulate transcription factors (TFs) such as ZEB1, SNAIL, and TWIST by integrating molecular mechanisms which are still not fully understood (1, 3, 4, 8, 9).

Several studies showed that ZEB1 (Zinc Finger E-box Binding Homeobox 1) has an active role in the induction of EMT in diverse epithelial malignancies, such as colon, prostate, pancreas, lung and breast cancer (10–15). Notably, the expression of ZEB1 in these tumors correlates with the loss of E-cadherin and is associated with advanced or metastatic disease, by triggering combined activation of cell motility and stemness properties (10, 16–18). However, there is still insufficient information regarding the signaling mechanisms that regulate ZEB1 during the EMT program. The regulation and function of ZEB1 in a physiological context has been widely studied (19–25).

Numerous investigations of recent years were focused in understanding the regulation of ZEB1 at different levels (transcriptional, translational, and post-translational). At transcriptional level, it has been reported that ZEB1 mRNA levels could be regulated by numerous TFs, among which SNAIL 1 and TWIST stand out because of their well-established participation in the regulation of the EMT program (26, 27). On a related note, numerous reports unveiled a role for miR-200 family members in the control of the epithelial phenotype through the regulation of ZEB transcription factors (ZEB1 and ZEB2). Interestingly, all miR-200 family members are direct transcriptional targets of ZEB1 and ZEB2, thus defining a double-negative feedback loop that control their mutual expression concomitantly with EMT progression (18, 28–32). At post-translational level, we have reported that changes in the phosphorylation status of ZEB1 modifies its binding to target promoters and ZEB1 transcriptional activity (33). In line with this, other research groups have shown that ZEB1 is a key TF during EMT induced by the TGF-β (34, 35). In addition, it has been reported that the IGF1 pathway is able to inducing and regulating the EMT program by regulation of ZEB1 levels in breast, prostate, endometrial and ovarian cancer, among others (11, 12, 36–38). Nonetheless, the molecular details and the regulatory players that orchestrate ZEB1 functionality during the EMT remain poorly understood today (39–44).

PKC isozymes play major roles in the control of signaling pathways associated with proliferation, migration, invasion, tumorigenesis, and metastasis, and have been associated with EMT (45–51). Based on their biochemical and structural properties, PKCs have been classified into three families: classical/conventional or calcium-dependent PKCs (cPKCs α, βI, βII, and γ), novel or calcium-independent PKCs (nPKCs δ, ε, ņ, and θ); and atypical PKCs (aPKCs ζ and ι). Despite their high homology and similar substrate specificity *in vitro*, PKC isozymes possess distinctive functional selectivity in cellular models due to their differential intracellular localization and access to substrates (46, 52–55). Changes in the expression levels or activation status of PKC isozymes have been reported in numerous human cancers, and in many cases correlations have been described between high levels of PKCs and degree of aggressiveness (47, 56, 57).

In this work, we unveiled an unforeseen role of PKCα in the upregulation of ZEB1 levels in breast cancer cells. Moreover, we also investigate the contribution of this PKCα/ZEB1 axis into the establishment and maintenance of the mesenchymal state and how PKCα-dependent modulation of ZEB1 levels controls tumor aggressiveness and metastasis using *in vitro* and *in vivo* models.

## Materials and Methods

### Cell Lines and Cell Culture

Cells used in this study were obtained from ATCC. MCF-10A cells were cultured in Dulbecco's Modified Eagle Medium/Nutrient Mixture F-12 (DMEM/F-12) (Thermo Scientific) supplemented with 5% equine serum (GIBCO), 1% penicillin-streptomycin (GIBCO), 20 ng/ml EGF (Sigma-Aldrich), 10 μg/ml insulin (Sigma-Aldrich), 0.5 mg/ml hydrocortisone (Sigma-Aldrich) and 100 ng/ml cholera toxin (Calbiochem). MCF-7 and T47-D cells were cultured in RPMI (GIBCO) supplemented with 10% fetal bovine serum (FBS; GIBCO), 1% L-glutamine (GIBCO) and 1% penicillin-streptomycin (GIBCO). NMuMG-NZEB1 and NMuMG-Vector cell lines were cultured in DMEM supplemented with 10% FBS, 1% L-glutamine, and 400 μg/ml G418 (Sigma-Aldrich). Other cell lines (HEK-293T; BT-549; MDA-MB-231; MDA-MB-468; SKBR-3; MDA-MB-361 and BT-474) were cultured in DMEM supplemented with 10% FBS and 1% penicillin-streptomycin. All the cell lines used in this work were negative for mycoplasma contamination.

### Stable Cell Lines Generation

NMuMG epithelial cells were transfected with eGFP-NZEB1 or eGFP-C3 empty vector (EV), using lipofectamine 2000 (Invitrogen) according to the manufacturer's instructions, followed by 10 days selection with geneticin (G418, Sigma-Aldrich). Two rounds of cell sorting for GFP-positive cells were performed after antibiotic selection (FACS Aria II, BD Bioscience). Stable knockdown of PKCα in MDA-MB-231 cells was achieved by transduction using the PLKO system of lentiviral shRNA-PKCα (Dharmacon) or shRNA-NTC as a control. Selection of stable cell lines was carried out with puromycin (2 μg/ml, Santa Cruz) for 10 days.

### DNA Constructs, shRNA, and RNAi

The full-length rat ZEB1 cDNA (21) was subcloned into pcDNA4/HisMaxB (Invitrogen) (ZEB1-FL). ZEB1 deletion mutants ZD1-HD and eGFP-NZEB1 were subcloned by into pcDNAI/Amp vector (Invitrogen) or eGFP-C3 (Clontech), respectively. Full-length ZEB1 and ZEB1 deletion mutants were a kind gift from Dr. Douglas S. Darling (University of Louisville, USA). The E-cadherin luciferase promoter was a kind gift from Dr. Frans Van Roy (University of Ghent, Belgium) (58). All constructs were verified by sequencing.

RNAi duplexes were purchased from Dharmacon (PKCα1: CCAUCCGCUCCACACUAAA; PKCα2: GAACAACAAGGAAUGACUU; PKCα3: UAAGGAACCACAAGCAGUA; PKCα4: UUAUAGGGAUCUGAAGUUA; PKCα5: GAAGGGUUCUCGUAUGUCA; PKCα6: UCACUGCUCUAUGGACUUA; ZEB1#1: CUGUAAGAGAGAAGCGGAA; ZEB1#2: CUGAAAUCCUCUCGAAUGA; ZEB1#3: GCGCAAUAACGUUACAAAU; ZEB1#4 GCAACAGGGAGAAUUAUUA; NTC: UGGUUUACAUGUUUUCUGA).

shRNAs were purchased from Dharmacon (PKCα: α1 TRCN1691; α2 TRCN1692; α3 TRCN1693) (ZEB1: Z1 TRCN17563; Z2 TRCN17565; Z3 TRCN17566), shNTC-pLKO.1 was obtained from Addgene (ID#1864).

### Transfections and Lentiviral Infection

RNAi duplexes (25 nM) were transfected using Lipofectamine RNAiMAX (Thermo Fisher Scientific). HEK-293T cells were transfected to obtain virus particles using JetPrime (Polyplus-transfection) as recommended by the manufacturer. Stable knockdown of PKCα in MDA-MB-231 was achieved by transduction using the PLKO system of lentiviral shRNA-PKCα (Dharmacon) or shRNA-NTC as a control according to the manufacturer's protocol.

### *In silico* Analysis

Prediction of potential ZEB1 phosphorylation sites was performed using by DISPHOS 1.3 KinasePhos and NetPhos 3.1 open source Web search tools (59–61).

### Luciferase Reporter Assays

HEK-293T cells (5 × 10^4^) were transfected by lipofection using PEI (PolyEthylenImine, Polysciences Inc.) (62). We used 0.3 μg of E-cadherin-Luc promoter and 0.3 μg of CMVβ clone (β-galactosidase reporter vector, Clontech) for normalization, which were co-transfected together with 0.5 μg of ZEB1-FL or each ZEB1 deletion mutant (ZD1-HD or NZEB1). Luciferase and β-galactosidase activities were evaluated as described (22). Results were expressed as the percentage of luciferase activity relative to the activity of the promoter with the empty vector (EV) (100%), normalized in each case to β-galactosidase activity.

### Treatment of Cells With Pharmacological Inhibitors

Cells were treated at the indicated times with the following inhibitors: GSK3 inhibitor LiCl (50 mM), Akt inhibitor LY294002 (20 μM, Calbiochem), MEK1/2 inhibitors PD98059 (20 μM, Calbiochem) and UO126, or its corresponding control UO124 (20 μM, Calbiochem), pan-PKCs inhibitor GF109203X (5 μM, Enzo Life Sciences) and Gö6983 (5 μM, Enzo Life Sciences), or the protein synthesis inhibitor cycloheximide (CHX, 25 μg/ml, Calbiochem). DMSO (Sigma-Aldrich) was used as vehicle and never exceeded a final concentration of 0.1%.

### Protein Analysis

Western blot analysis (WB) was carried out essentially as previously described in (63). The detection and quantification were performed with Odyssey Clx System (LI-COR Biosciences) through the Image Studio Software, or by traditional ECL detection. The following antibodies were used: anti-ZEB1-1642 (from Dr. Douglas Darling Lab), anti-ZEB1 (Santa Cruz,# sc-25388), anti-E-cadherin (BD Biosciences,# 610182), anti-vimentin (Cell Signaling, #5741), anti-SNAIL (Cell Signaling, #3879), anti β-catenin (Cell Signaling, #8480), anti ZO-1 (Cell Signaling, #8193), anti-cytokeratin 18 (Cell Signaling, #4548), anti-phospho serine/threonine (Abcam, #ab9337), anti-phospho-substrates antibodies kit Cell Signaling, #9920) anti-GFP (Abcam, #ab290), anti-PKCα (Santa Cruz, # sc-208), anti-PKCδ (Cell Signaling, #2058) anti-PKCε (Santa Cruz,# sc-1681), anti- Phospho-PKCα/β II (Cell Signaling, #9375) anti-α-tubulin (Sigma-Aldrich, #T5168); anti-β-actin (Sigma-Aldrich, #A2228); anti-PCNA (Santa Cruz, #sc56). As secondary antibodies we used anti-rabbit-Alexa-Fluor 594 (Molecular Probes), anti-rabbit HRP (Cell Signaling, #7074), anti-mouse HRP (Cell Signaling, #7076), goat anti-mouse IRDye 680RD and goat anti-rabbit IRDye 800CW (LI-COR Biosciences).

### Immunoprecipitation (IP)

HEK-293T cells were transfected with either ZD1-HD or NZEB1, or the corresponding empty vectors (pcDNA1 or eGFP). After 48 h, cells were lysed in RIPA buffer. Cell extracts were subject to IP using either anti-ZEB1-1642 (from Dr. Douglas Darling Lab) or anti-GFP (Abcam, #ab290). An anti-IgG antibody was used as control. IP assays were performed using the Classic IP kit (Pierce) according to the manufacturer's instructions. Eluted immune-complexes were run in 10% SDS-PAGE gels and Western blot analysis was performed using the indicated antibodies.

### Pull-Down With Ni2+ Beads

HEK-293T cells were co-transfected with His6-ZEB1 (HM-ZEB1) and untagged PKCα for 48 hs. Before pull-down, cells were pretreated with PMA 10 nM for 15 min. The purification was carried out under non-denaturing conditions. The pull-down assay was performed using Ni-NTA Agarose (QIAGEN) according to the manufacturer's instructions. Eluted immune-complexes were resolved in 10% SDS-PAGE gels and Western Blot were performed using the indicated antibodies.

### Real-Time Quantitative PCR (RT-qPCR)

Total RNA from cells was isolated using the RNeasy Mini Kit (Qiagen). One μg of total RNA was reverse transcribed to cDNA with the TaqMan Reverse Transcription Kit (Invitrogen). qPCR was performed in a ABI PRISM 7700 detection system using TaqMan Universal PCR MasterMix (Applied Biosystems), target primers (900 nM), fluorescent probe (250 nM) and cDNA. TaqMan probes specific for PKCα, ZEB1, E-cadherin, vimentin and the housekeeping gene UBC (used for normalization) were purchased from Applied Biosystems (ZEB1: ZEB1-FAM™ dye Hs01566408_m1; PKCα: PRKCA-FAM™ dye Hs00925195_m1; vimentin: Vimentin-6FAM™ dye Hs00185584_m; E-cadherin: E-cad-6FAM™ dye Hs01023894_m1; UBC: UBC-VIC™ dye Hs00824723_m1). PCR product formation was continuously monitored using the Sequence Detection System software version 1.7. Results were expressed as fold-change of the target gene by the 2-ΔΔCt method and normalized to the NTC sample. All qPCR reactions were performed in triplicate. Every experiment was independently performed three times.

### Immunostaining and Confocal Microscopy

Cells growing on glass coverslides were fixed with 4% paraformaldehyde, stained with an anti-E-cadherin antibody anti-E-cadherin (BD Biosciences, # 610182) or rhodamine-phalloidin (Molecular Probes), and nuclei counterstained with DAPI (0.3 μg/ml, Sigma-Aldrich). Confocal images were taken with an Olympus FluoView FV1000 Laser Scanning confocal microscope.

### Wound Healing Assay

Cells were grown to full confluence in 6-well plates in DMEM and reduced serum supplementation (1%). Subsequently, a wound was made using a pipette tip, and the plate was washed twice with media to remove detached cells. Photomicrographs of initial wounds (t:0) and final wounds after 16 or 24 h (t:16 or t:24) were captured using an optical microscope equipped with a motorized stage (Leica DMI 8). The initial and final wound areas were measured using the ImageJ/Fiji software, and the difference between the two was used to determine migrated area. Experiments were done in triplicate, and every experiment was performed independently three times.

### Invasion Assay

Cells were trypsinized, suspended in 0.1% BSA/DMEM, and seeded (2.5 × 10^4^ cells/well) in the upper compartment of a Boyden chamber (NeuroProbe). Matrigel-coated polycarbonate membranes (8-μm pore diameter) were used to separate the upper and lower compartments. In the lower chamber, DMEM medium containing 10% FBS was used. After an incubation period of 16 or 24 h at 37°C, membranes were recovered and cells on the upper side of the membrane (non-invasive) were wiped off the surface. Cells on the lower side of the membrane (invasive) were fixed and stained with the Hema 3 Staining kit (Thermo Scientific). Invasive cells in each well were counted in five random fields by contrast microscopy using an optical microscope equipped with a motorized stage (Leica DMI 8) and the ImageJ/Fiji software. Each condition was assessed by triplicate. Every experiment was performed independently three times.

### Soft Agar Colony Formation Assay

The soft agar colony formation assay in NMuMG cells (NZEB1 or EV) was carried out as a previously described in (64). Ten independent fields were photographed using an optical microscope (NIKON ECLIPSE TE2000-U). The quantification of the number of colonies formed by more than 20 cells was performed. Each condition was assessed by triplicate. Every experiment was performed independently three times.

### *In vivo* Experimental Metastasis Assay

All animal experimental protocols were approved by the Ethical Committee on Animal Care and Use (CICUAL 63/2016), University of Buenos Aires (Argentina), and experiments performed in compliance with the ARRIVE Animal Research guidelines recommendations. Six to eight-week-old NOD/SCID male mice were inoculated with 27 G needles containing 1 × 10^6^ cells through tail vein injection. Animals were housed with access to food and water *ad libitum* in ventilated mouse cages (1–5 mice per cage) at the IBioBA Institute Animal Services Facility and were randomly divided into the different treatment groups. Seventy-five days after injection, animals were euthanized and their lungs were carefully inflated with a neutralized buffered formalin (NBF) solution containing 40% formalin, 4 g/liter NaH2PO4, and 6.5 g/liter Na2HPO4. Lungs were then removed and collected into NBF containing tubes to enable 24 h fixation at room temperature. Tissues were then washed and stored in a 70% ethanol solution.

### Metastatic Foci Analysis

Lung tissue DNA sample preparation for metastatic foci analysis was performed with Qiagen DNeasy® Blood & Tissue kit, following the manufacturer's instructions. qPCR was performed to determine the relative abundance of human DNA in mice tissues. The amplification reactions of 40 cycles were carried out with specific primers for human GAPDH (Fw: 5′ TCGACAGTCAGCCGCATCTTCT 3′, Rv: 5′ GAACACATCCGGCCTGCGC 3′) and mouse GAPDH (Fw: 5′ ATGCCACCGACCCCGAGGAA 3′, Rv: 5′ CCTGGCGATGGCTCGCACTT 3′) using FastStart Essential DNA Green Master Mix from Roche. Data were analyzed with Bio-Rad CFX M. For each sample, the values were normalized to mouse GAPDH levels. The Human GAPDH /Mouse GAPDH ratio was used to quantify the incidence of metastasis.

### *In silico* Analysis of PRKCA and ZEB1 mRNA Expression Among Breast Cancer Cell Lines

Pre-processed PRKCA and ZEB1 mRNA expression levels and intrinsic subtypes among 54 breast cancer cell lines were obtained from the Heiser 2012 dataset (E-MTAB-181) at UCSC Xena browser (https://xenabrowser.net/). Univariate and bivariate Pearson's test was employed for correlation analysis between both genes using R software.

### Statistical Analysis

Statistical analysis was performed using GraphPad Prism 6.0 (GraphPad Software), applying two-tailed Student's *t*-test or analysis of variance (ANOVA) test as appropriate. A *p* < 0.05 was considered statistically significant. Statistical probability is expressed as ^*^p < 0.05, ^**^p < 0.01, and ^***^p < 0.001.

## Results

### A Small N-Terminal Fragment of ZEB1 That Retains Its Capacity to Promote the EMT Is a PKC Substrate

**Figure 1:**
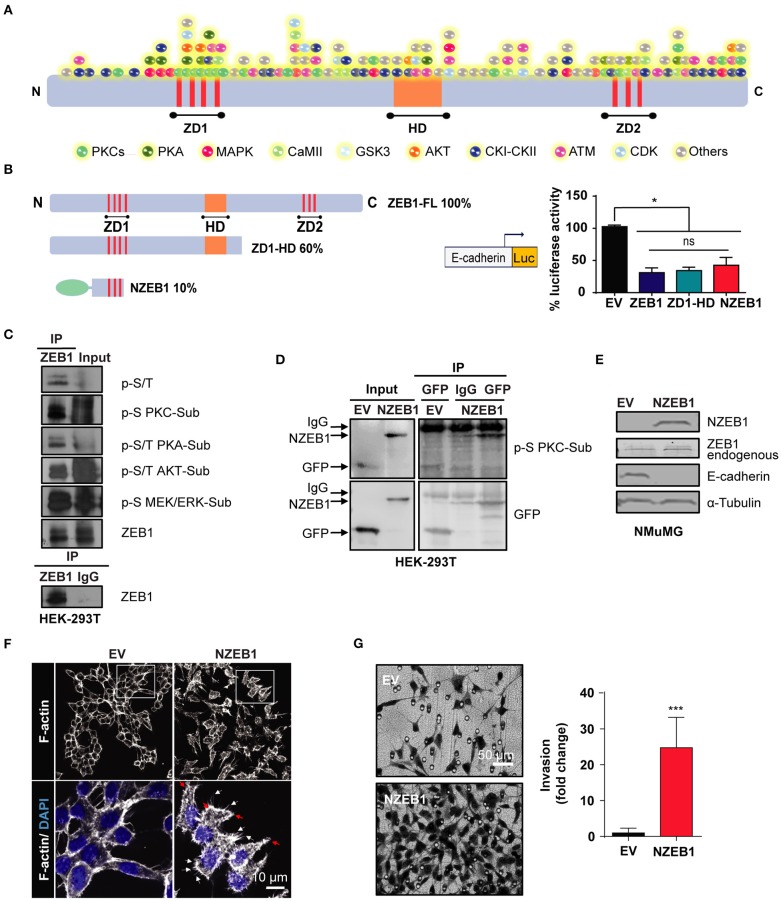
An N-terminal fragment of ZEB1 which retains its EMT-inducing capacity is a phospho-substrate of PKCs. **(A)** Scheme of the *in-silico* analysis of ZEB1 potential phosphorylation sites. **(B)** Left: schematic representation of ZEB1 full-length (*ZEB1-FL*) and the deletion mutants used: ZD1-HD and NZEB1 (*ZD1*: N-terminal zinc finger cluster, *HD*: Homeodomain, and *ZD2*: C-terminal zinc finger cluster). Right: Luciferase reporter assay of the E-cadherin promoter activity in HEK-293T cells co-transfected with either ZEB1-FL or ZEB1 deletion mutants. **(C,D)** Immunoprecipitation of ZD1-HD or NZEB1. Western blots were performed using a specific set of anti-phospho substrates antibodies for MEK/ERK (p-S MEK/ERK-Sub), PKCs (p-S PKC-Sub), PKA (p-S/T PKA-Sub) and AKT (p-S/T AKT-Sub) kinases; anti-phospho serine/threonine (p-S/T), anti-ZEB1 or anti-GFP. **(E)** Western blots for endogenous ZEB1, NZEB1 and E-cadherin in NMuMG epithelial cells stably expressing NZEB1 or GFP-Empty Vector (EV). **(F)** Confocal microscopy analysis of F-actin cytoskeleton distribution determined by rodamine-phalloidin staining (*white*) in stable NZEB1 or EV NMuMG cell lines. Nuclei were stained with DAPI (*blue*). Magnification: 60X; Bar: 10 μm. Red and white arrows indicate lamellipodia and filopodia, respectively. **(G)** Matrigel invasion assay in NZEB1 or EV NMuMG cells. Magnification: 10X. Bar: 50 μm. Results are expressed as mean ± S.D>. *ns*, not significant, *p ≤ 0.05, ***p ≤ 0.001).

While changes in the phosphorylation status of ZEB1 are known to affect its binding to gene promoters and transcriptional activity, the kinases responsible for ZEB1 phosphorylation have not been yet described. As a first approach to identify putative ZEB1 kinases in cancer cells, we performed an *in-silico* screening of phosphorylation sites for multiple central kinases, including PKCs, PKA, MAPKs, and AKT. For this analysis, we used DISPHOS 1.3 KinasePhos and NetPhos 3.1 open source web search tools, which revealed a large number of potential phosphorylation sites in ZEB1. Even focusing on those sites that display high scores, there were a plethora of putative sites distributed in all ZEB1 functional domains (ZD1, HD, and ZD2) and across the rest of the protein, as highlighted in Figure 1A. Due to this level of complexity, we concluded that a classical site-directed mutagenesis would be an unsuitable experimental strategy to identify novel regulatory kinases for ZEB1. Thus, we decided to follow up the analysis by using ZEB1 deletion mutants (ZD1-HD and NZEB1). These constructs represent 60% and 10% of the full-length protein, respectively, and both retain the capacity to repress the E-cadherin promoter in cells, as determined with a luciferase reporter assay in cells (Figure 1B). To explore the involvement of regulatory kinases identified in the *in-silico* screening, we analyzed the phosphorylation of ZEB1 using a panel of commercial phospho-substrate antibodies against consensus sites of various kinases, namely MEK/ERK, PKC, AKT, and PKA, as well as with an anti-phospho serine/threonine antibody. Immunoprecipitation experiments were performed in HEK-293T cells and, as expected from the *in-silico* analysis, we found that ZEB1 is a substrate of multiple kinases in cells (Figure 1C).

A closer look to the NZEB1 sequence revealed a remarkable enrichment in phospho-sites for PKCs (Figure 1A). To determine if NZEB1 can be indeed phosphorylated by PKC in cells, we expressed it as a GFP-fused protein in HEK-293T cells, and immunoprecipitated the protein with an anti-GFP antibody. Notably, using an anti-PKC phospho-substrate antibody, we found significant immunoreactivity in the immunoprecipitates against GFP-NZEB1 but not against GFP alone (Figure 1D). Based on these preliminary findings, we decided to focus our study on a potential regulatory link between ZEB1 and PKC.

Next, we wished to confirm that GFP-NZEB1 represented a suitable model to study the regulation of ZEB1. Toward this goal, we investigated if GFP-NZEB1 recapitulates the phenotypes observed during the activation of the EMT program, as normally observed with the full-length protein. A stable cell line expressing GFP-NZEB1 was derived from NMuMG mammary epithelial cells (Supplementary Figure 1A). We evaluated the levels of several EMT markers (Figure 1E and Supplementary Figure 1B). We found that stable expression of GFP-NZEB1 repressed the expression of epithelial hallmark E-cadherin (Figure 1E and Supplementary Figure 1C), affecting also the expression and subcellular localization of β-catenin (Supplementary Figures 1B,D) and increasing the expression of the mesenchymal marker Vimentin (Supplementary Figure 1B). These changes in the expression of EMT markers correlated with the loss of the epithelial phenotype and the acquisition of mesenchymal traits (Supplementary Figure 1E), which are characterized by a dramatic change in the cell-cell interactions, overall cellular shape, and the reorganization of the cortical actin cytoskeleton (Figure 1F). Moreover, the acquisition of the mesenchymal phenotype was accompanied by increased migratory capacity (Wound healing—Supplementary Figure 1F), invasiveness (Matrigel invasion assay—Figure 1G) and anchorage-independent cell growth (Soft agar colony formation—Supplementary Figure 1G).

### PKCα Levels and Activity Correlate With ZEB1 Levels in Breast Cancer Cells

**Figure 2:**
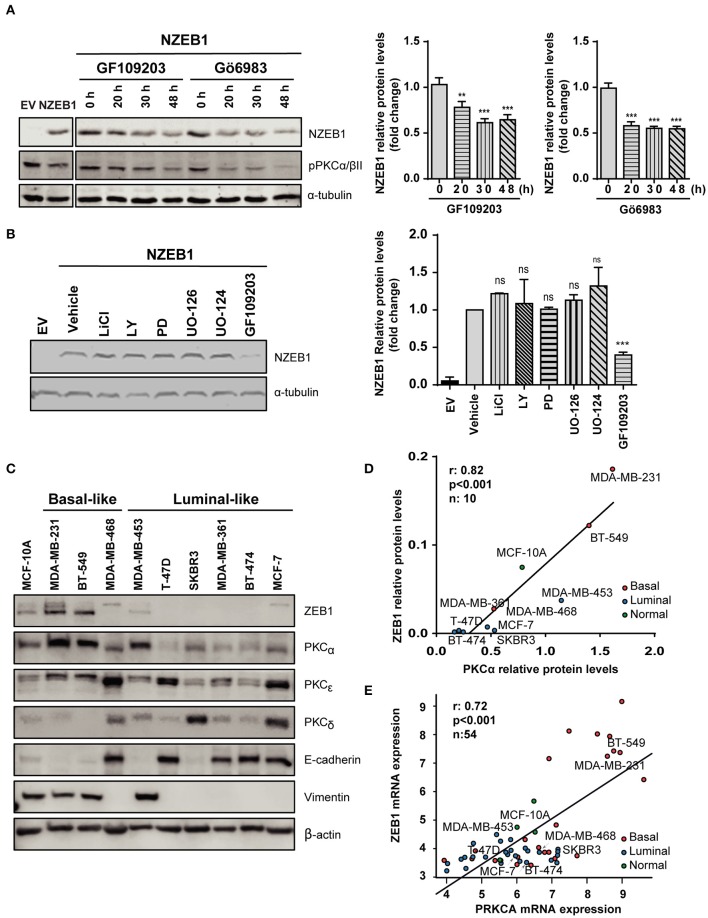
PKCα levels and activity correlate with ZEB1 levels in breast cancer cells. Western blot analysis of NZEB1 levels in NMuMG-NZEB1 cells **(A)** treated with the pan-PKC inhibitors GF109203X (*GF*) or GÖ6983 (*GÖ*) (5 μM) for the indicated times; or **(B)** treated with specific inhibitors of several signal transduction pathways: GSK3 inhibitor LiCl (50 mM), AKT inhibitor LY294002 (*LY*, 20 μM), MEK1/2 inhibitors PD98059 (*PD*, 20 μM) and UO126 and its respective negative control UO124 (20 μM), pan-PKCs inhibitor GF109203X (*GF*, 5 μM) or DMSO (vehicle) for 48 h. Graphs represent protein levels of NZEB1 (fold-change), normalized to the loading control and relativized to time 0 h in **(A)**, or to DMSO (vehicle) in **(B)**. Results are expressed as mean ± S.D. **p ≤ 0.01; ***p ≤ 0.001). **(C)** Western blot analysis of ZEB1, PKCα, PKCε, PKCδ, and EMT markers (E-cadherin and vimentin) in breast cancer cells. **(D)** Analysis of protein expression levels of PKCα and ZEB1 in the entire set of breast cancer cell lines. Pearson *r* = 0.82. ***p ≤ 0.001. **(E)** *In silico* analysis of PKCα and ZEB1 mRNA expression among 54 breast cancer cell lines in the Heiser 2012 dataset. Pearson *r* = 0.72. ***p ≤ 0.001).

Based on our previous results, we investigated a potential functional regulation of ZEB1 by PKC. NMuMG cells stably expressing NZEB1 were treated with the pan-PKC inhibitors GF109203X or Gö6983. Interestingly, WB analysis revealed that NZEB1 levels decreased in response to both PKC inhibitors, whose activities were confirmed by a reduced phosphorylation of PKCα/βII (Figure 2A). On the other hand, no effect on ZEB1 expression could be detected when cells were treated with the GSK3 inhibitor LiCl, the PI3K inhibitor LY294002, or the MEK1/2 inhibitors PD98059 and UO126. These results uncover a specific role of PKC in the modulation of ZEB1 protein expression levels, and are consistent with the lack of relevant phosphorylation sites for GSK, Akt, and MEK/Erk within NZEB1 (Figure 2B).

To explore in more detail the link between PKC and ZEB1, we investigated a panel of breast cancer cells of different subtypes (luminal and basal-like) and different degrees of aggressiveness. We found that basal-like breast cancer cell lines express high ZEB1 levels (particularly MDA-MB-231 and BT-549 cells). These cells also express high levels of PKCα and display mesenchymal features (low E-cadherin, high vimentin). On the other hand, luminal cells generally express low ZEB1 levels, and in most cases display lower expression of PKCα compared to basal-like breast cancer cells (with the exception of MDA-MB-453 cells (Figure 2C). Indeed, a positive correlation between ZEB1 and PKCα expression levels was found for the different breast cancer cell lines used in this analysis (*r* = 0.82, *p* < 0.001) (Figure 2D). Such correlation was not observed for other PKC isozymes known to have important roles in breast cancer progression, namely PKCδ and PKCε (45) (Supplementary Figures 2A,B). To expand on this finding, we bioinformatically explored the Heiser 2012 dataset (E-MTAB-181), where we cross-examined the mRNA levels of ZEB1 and PKCα in a larger panel of breast cancer cell lines. This analysis also revealed a significant positive correlation between ZEB1 and PKCα protein expression levels, not only for the cell lines described in Figure 2D but also for the entire panel comprising 54 breast cancer cell lines (*r* = 0.72, *p* < 0.001) (Figure 2E). These results strongly suggest a link between PKCα activity and ZEB1 that potentially contributes to EMT in breast cancer cells.

### PKCα Knockdown Reduces Endogenous ZEB1 Levels in Breast Cancer Cells

**Figure 3:**
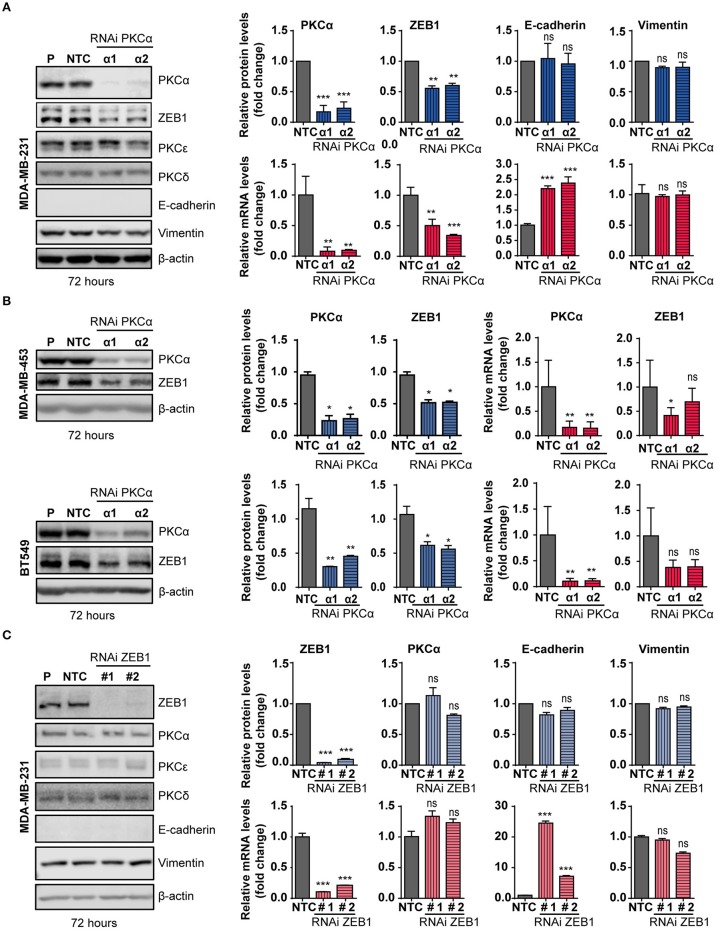
PKCα silencing or inhibition downregulates ZEB1 levels. MDA-MB-231 **(A)**, MDA-MB-453 and BT549 **(B)** cells were transfected with specific PKCα RNAi duplexes (α1 and α2) or the non-target control RNAi (*NTC*). Western blot analysis for PKCs and EMT markers was carried out 72 h later. *P*, parental cells. Blue bar graphics represent protein levels (fold-change) normalized to β-actin and relativized to the NTC. mRNA levels of PKCα, ZEB1, E-cadherin and vimentin **(A)** or PKCα and ZEB1 **(B)** were assessed by RT-qPCR and results were plotted on the red bar graphics (fold-change) normalized to the housekeeping gene UBC and relativized to NTC. **(C)** MDA-MB-231 cells were transfected with specific RNAi duplexes for ZEB1 (#1 and #2) or NTC RNAi. Western blot analysis for PKCs and EMT markers was carried out 72 h later. The graphics (blue bars) represent the quantification (fold-change) normalized to β-actin and relativized to the NTC. PKCα and ZEB1 mRNA levels were represented on the red bar graphics as fold-change, normalized to the UBC and relativized to the NTC. Results are expressed as mean ± S.D. *ns*, not significant; *p ≤ 0.05; **p ≤ 0.01; ***p ≤ 0.001.

The observed correlation between PKCα and ZEB1 in breast cancer cell lines prompted us to explore a direct link between them in more detail. We focused on MDA-MB-231 breast cancer cells, a model that shows high levels of expression of ZEB1 and PKCα, and in which ZEB1 function has been extensively studied (48, 65, 66). To determine whether a causal relationship exists between PKCα and ZEB1 expression levels, we silenced PKCα using RNAi. Two different duplexes (α1 and α2) were used, which reduced PKCα mRNA levels by >95% and PKCα protein levels by >80% at 72 h post-transfection (Figure 3A). Interestingly, upon PKCα silencing, endogenous ZEB1 mRNA and protein levels were significantly reduced (Figure 3A). In the time-frame of these experiments, the observed ZEB1 down-regulation was not sufficient to trigger a detectable induction in E-cadherin protein levels, yet a significant increase in E-cadherin mRNA levels was observed, consistent with a reversion of the mesenchymal phenotype taking place (Figure 3A). These results were validated using 4 additional siRNAs sequences for PKCα (Supplementary Figure 3A), as well as by pharmacological inhibition with GF109203X or Gö6983 (Supplementary Figure 3B). Moreover, ZEB1 protein levels were also consistently reduced in MDA-MB-453 and BT-549 cells upon PKCα silencing, although ZEB1 mRNA decrease in these cell lines was not as substantial as the one observed in MDA-M-231 cells (Figure 3B).

Data from Figure 3, together with the experiments shown in Figure 2 with NZEB1 (which is expressed from an exogenous promoter), suggest that ZEB1 might be downregulated both at mRNA and protein levels upon PKCα silencing. To explore this scenario, we performed experiments using the protein synthesis inhibitor cycloheximide (CHX) and examined ZEB1 half-life. Cell were transfected with PKCα RNAi duplexes and 60 h later treated with CHX for 12 or 16 h. We found that CHX-treated cells have reduced ZEB1 protein levels when PKCα was inhibited (Supplementary Figure 3C). A similar result was observed when PKC was pharmacologically inhibited by GF109203X for 48 h (Supplementary Figure 3C). Together, these findings suggest the co-existence of transcriptional and post-transcriptional mechanisms that promote ZEB1 downregulation in response to PKCα inhibition.

To explore the hierarchy within the PKCα/ZEB1 axis, we analyzed the expression of PKCα in MDA-MB-231 cells after ZEB1 silencing. In this scenario, we found no evidence of changes in the expression of PKCα or other PKCs isozymes in ZEB1-KD cells. Note that at 72 h post-transfection, there is no E-cadherin protein up-regulation (despite elevation in E-cadherin mRNA levels) or changes in vimentin expression (Figure 3C). However, an elevation in E-cadherin protein levels becomes obvious at 96 h, and using 4 different ZEB1 RNAi sequences (Supplementary Figure 3D). Thus, while PKCα has an important upstream role in regulating ZEB1 levels, ZEB1 does not control the expression of PKCα. Interestingly, by performing immunoprecipitation experiments of exogenous ZEB1 we were able to pull-down PKCα (Supplementary Figure 3E), which further supports a mechanism of direct regulation of ZEB1 stability by PKCα phosphorylation.

### PKCα Knockdown Recapitulates EMT Inhibition and Modulates the Metastatic Potential of Breast Cancer Cells *in vivo*

**Figure 4:**
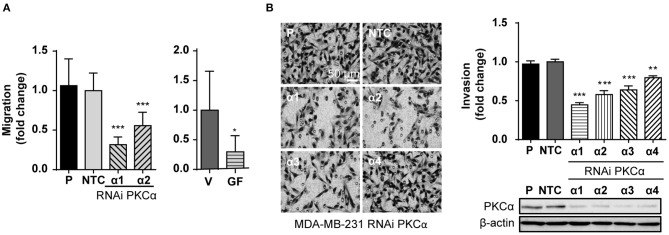
PKCα silencing recapitulates the EMT phenotypes associated with ZEB1 downregulation. **(A)** MDA-MB-231 cells were transfected with specific PKCα RNAi duplexes (α1 and α2) or NTC RNAi for 72 h or treated with GF109203X (*GF*, 5 μM) for 48 h. Migration was assessed using a wound healing assay. Graphics represent the migrated area relativized to NTC or vehicle (V), respectively. **(B)** MDA-MB-231 cells were transfected with four different PKCα RNAi duplexes (α1, α2, α3, and α4) or NTC RNAi, and invasion was assessed 72 h later. The images display representative fields of invading cells. Bar: 50 μm. Graphs show the percentage of invading cells per field relativized to the NTC. Results are expressed as mean ± S.D. *p ≤ 0.05; **p ≤ 0.01; ***p ≤ 0.001.

An immediate question that arose from exposing the link between PKCα and ZEB1 levels was if PKCα downregulation is sufficient to recapitulate the reversion of the mesenchymal phenotype observed after ZEB1 knockdown. Given the relevance of ZEB1 in cancer progression, we were particularly interested in exploring if the migratory and invasive capacities of breast cancer cells were altered upon PKCα RNAi depletion, similar to when ZEB1 is down-regulated (Supplementary Figures 4A,B). Notably, we found that the migratory capacity of MDA-MB-231 cells was markedly reduced either upon PKCα silencing or GF10923X treatment, as determined using a wound healing assay (Figure 4A). Likewise, when we used the Matrigel invasion assay, we unveiled also an important reduction in the invasive capacity of cells subjected to PKCα knockdown (Figure 4B).

**Figure 5:**
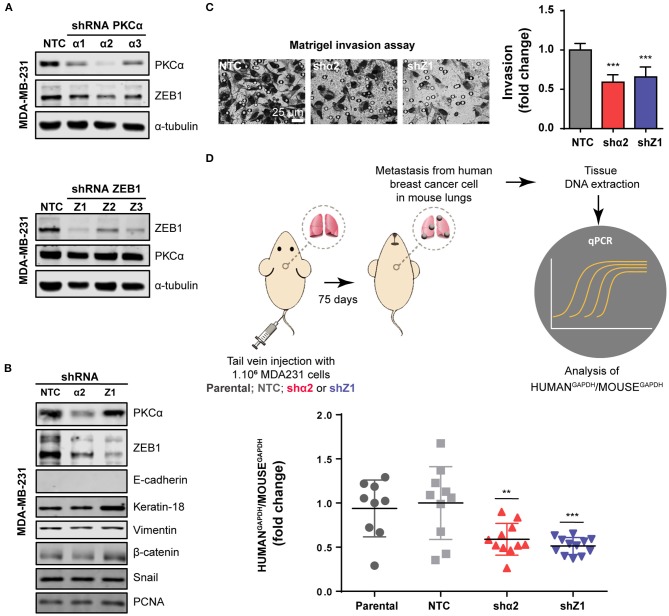
PKCα is required for breast cancer cell metastasis. **(A)** Stable knockdown of PKCα (shα1, shα2, shα3) or ZEB1 (Z1, Z2, Z3) in MDA-MB-231 cells was achieved using the PLKO lentiviral system. **(B)** Western blot analysis for EMT markers. **(C)** Invasiveness was assessed by the Matrigel invasion assay. Graphics represent the number of invading cells per field relativized to NTC. **(D)** Scheme of the experimental metastasis assay in NOD/SCID mice. Seventy-five days after cell injection, murine, and human levels of GAPDH were analyzed by RT-qPCR in mouse lungs. Graphs show the ratio of Human GAPDH /Mouse GAPDH relativized to the NTC control. Results are expressed as mean ± S.D. **p ≤ 0.01; ***p ≤ 0.001.

Finally, given the relevance of EMT in metastasis, we extended our findings to an *in vivo* model. To this aim, we used a mouse model of experimental metastasis that involves the tail injection of MDA-MB-231 cells and the quantification of metastatic cells that colonize the lungs. Since this is long-term protocol (2.5 months), we used a stable knockdown approach. Using the PLKO system of lentiviral shRNA expression, we transduced MDA-MB-231 cells with three different shRNAs against PKCα or ZEB1, followed by selection with puromycin. Importantly, in MDA-MB-231 cells subjected to stable PKCα depletion we observed a concomitant downregulation of ZEB1 (Figures 5A,B), thus recapitulating our findings using transient RNAi and PKC inhibitors (see Figure 3 and Supplementary Figure 3). Along the same line, ZEB1 stable depletion did not alter PKCα levels. Notably, we found that the invasive capacity of MDA-MB-231 cells was markedly reduced upon stable depletion of either PKCα or ZEB1 (Figure 5C), as we previously observed with RNAi and PKC pharmacological inhibitors (see Figure 4 and Supplementary Figure 4).

For the experimental metastasis experiment we selected the stable cell line with the strongest PKCα downregulation (shRNAα2) and ZEB1 (shRNA#1). To precisely quantify colonization of mouse lungs by the breast cancer cells, we used a protocol were the ratio of Human/Mouse cells is determined by q-PCR using primers for human and mouse GAPDH, respectively (Human GAPDH /Mouse GAPDH) (Figure 5D). Strikingly, we observed that PKCα downregulation impaired the metastatic capacity of breast cancer cells, as we also observed for ZEB1 knockdown (Figure 5D). Taken together, these findings demonstrate that an axis comprising PKCα-ZEB1 modulates the metastatic potential of breast cancer cells by promoting multiple mesenchymal features during the progression of the disease.

## Discussion

The activation of EMT has a documented role in promoting a metastatic phenotype (8, 9). While EMT might be important in essentially all types of cancers known to metastasize (4, 7, 9, 67), it becomes particularly relevant in breast carcinomas. This is due not only to their epithelial origin, but also to the fact that *in situ* breast carcinomas do not pose a major life threat for patients, in contrast to primary tumors on other body locations such as the brain or other internal organs (68–70). In fact, the transformation to an invasive state and the subsequent metastasis and colonization of distal organs is the main cause of death in breast cancer patients (68, 69, 71, 72).

In this work we have approached the question of how the activity of the EMT transcription factor ZEB1 can be modulated in breast cancer cells. Using an unbiased approach to find phospho-sites in ZEB1, we identified a previously unknown relationship between this transcription factor and PKCα, a kinase widely implicated in tumorigenesis and metastasis (45, 48, 66).

### PKCα Is a Key Regulator of ZEB1 Expression Levels in Breast Cancer Cells

Our study builds up on the central role of ZEB1 during the EMT in mammary cells, being able to activate several EMT features such as E-cadherin down-regulation and the acquisition of a highly invasive phenotype required for metastasis. These findings feed the current controversy of whether the EMT is dispensable or indispensable for the metastatic phenotype of cancer cells (10, 17, 72–74). The findings reported herein support the notion that ZEB1 is an essential player of the metastatic phenotype in breast cancer cells. Notably, a deletion mutant comprising only 10% of full length ZEB1 was sufficient to trigger a consistent EMT activation in non-transformed NMuMG mammary epithelial cells. This truncated protein is indeed heavily phosphorylated by PKCs, as determined *in silico* and with an anti-phospho-PKC substrate motif antibody.

A key finding of this study is that ZEB1 is highly expressed in basal-like breast cancer cells, which are known to possess a high metastatic potential (68, 69, 72). A strong correlation was observed between ZEB1 and PKCα levels in breast cancer cells, with a remarkable high expression of ZEB1 and PKCα in MDA-MB-231 and BT-549 basal-like cells, which display a significant degree of aggressiveness. The causality of this association was further confirmed using PKCα silencing approaches, which triggered a pronounced downregulation of ZEB1 in several breast cancer cell lines. These findings not only unveil a previously unforeseen regulatory pathway whereby PKCα controls ZEB1 expression levels, but also consolidates PKCα as a central player in the EMT program. Such notion has been put forward before by different lines of evidence. First, seminal work from the Weinberg's lab showed that transformation of normal mammary cells induced by EMT transcription factors TWIST, SNAIL, and SLUG triggers a consistent up-regulation of PKCα (48). Second, PKCα overexpression in MCF-7 and T-47D cells was found to promote anchorage-independent growth and the loss of the epithelial phenotype (75, 76). More recently, PKCα has been linked to elevated p120-catenin and claudin-1 levels (77), two markers of activated EMT program. In addition, a recent study reported that PKCα stabilizes TWIST1 expression (78). Taken together, these results argue for a central role for PKCα in the control of EMT by regulating of the expression of transcription factors.

An outstanding question that remains to be addressed in future studies is the relevance of the PKC-ZEB1 pathway in other types of human cancers. Even though in this study we focused on breast cancer due to our initial findings with a collection of breast cancer cells (Figure 2), there is no reason to assume that a similar scenario could not be observed in other types of cancers as well. In fact, ZEB1 has been implicated in the promotion of metastasis in other malignancies such as ovarian (79), gastric (80), pancreatic (10, 81), and hepatocellular carcinoma (82).

Another important point to consider in future studies relates to the nature of the underlying mechanism by which PKCα regulates ZEB1 expression. Our results argue for the existence of multiple mechanisms, which may not necessarily apply to every breast cancer cell line. For instance, PKCα RNAi depletion strongly reduced ZEB1 mRNA levels in MDA-MB-231 cells, but to a lesser extent in BT-549 cells and MDA-MB-453 cells. These differences may reflect different penetrance among cell types. Nonetheless, database analysis revealed significant correlation in a set of 54 breast cancer cell lines, suggestive of conserved regulatory mechanisms among breast cancer cells at the mRNA level. Our studies also suggest additional PKCα-regulated post-transcriptional mechanisms that control ZEB1 protein stability, which might not be mutually exclusive with those controlling mRNA expression. Indeed, regulation of ZEB1 protein stability via phosphorylation has been previously described, such as ATM-mediated ZEB1 phosphorylation and upregulation in response to DNA damage (83). Other EMT transcription factors, such as SNAIL and TWIST1, are also subject to phosphorylation-mediated stabilization (42). The identification of the post-translational mechanisms controlling ZEB1 stability is beyond the goals of the present study. Since ZEB1 is subject to ubiquitination (84), we speculate that PKCα may control ZEB1 stability via regulation of ubiquitin ligases or other components of the proteasomal degradation machinery. Another intriguing possibility is that the miR-200 family of microRNAs, which has been linked to the expression control of both ZEB1 and PKCα (18, 32, 85, 86) might play a role in the regulation of the PKCα-ZEB1 axis.

### Inhibition of the EMT as a Therapeutic Strategy: A Potential Niche for PKC Inhibitors?

The intuitive concept of targeting metastasis has been around in the oncology field for a long time. However, in comparison to classical cytotoxic therapies, target discovery for the exclusive inhibition of metastasis is far behind (87). Metastatic dissemination could be tackled at multiple steps, from the detachment of the cancer cells from the primary tumor to the nesting at distant sites (88). Herein, we provide compelling evidence indicating that the inhibition of PKCα could be used as a therapeutic strategy to impair breast cancer cell invasion and metastasis. Based on our results, PKCα represents an attractive approach to translate into a clinical setting. PKCα inhibitors have been generated and assessed as anti-tumor agents (46–48, 89–95). Moreover, pharmacological inhibition of PKCα inhibits the growth of xenografts derived from triple-negative breast cancer patients, which express high PKCα levels, but also selectively target cells that have undergone EMT, which are also enriched for stem cell properties (48). Taken together, it would be interesting to thoroughly assess the use of selective PKCα inhibitors as anti-cancer agents in triple-negative breast cancer. Our results also highlight the potential of repositioning PKCα inhibitors for their use as antimetastatic agents.

## Data Availability Statement

All datasets generated for this study are included in the article/Supplementary Material.

## Ethics Statement

The animal study was reviewed and approved by CICUAL 63/2016.

## Author Contributions

ML, MK, and GS: conceptualization. ML, FR, IG, MC, MA, CL-H, LB-R, and MV: methodology. ML, FR, and IG: investigation. MC, MK, and GS: writing-original draft. JB, MR, MK, and GS: supervision. GS: project administration. JB, MR, MK, and GS: funding acquisition. All authors contributed to manuscript revision, read, and approved the submitted version.

### Conflict of Interest

The authors declare that the research was conducted in the absence of any commercial or financial relationships that could be construed as a potential conflict of interest.
