# Supplementary material for: PKCα Modulates Epithelial-to-Mesenchymal Transition and Invasiveness of Breast Cancer Cells Through ZEB1
Source: Front Oncol. 2019 Nov 27;9:1323. doi: 10.3389/fonc.2019.01323 (PMC6890807; doi:10.3389/fonc.2019.01323)
Supplement: Supplementary file 1 [file Presentation_1.pdf]

Supplementary Material

Supplementary Figures

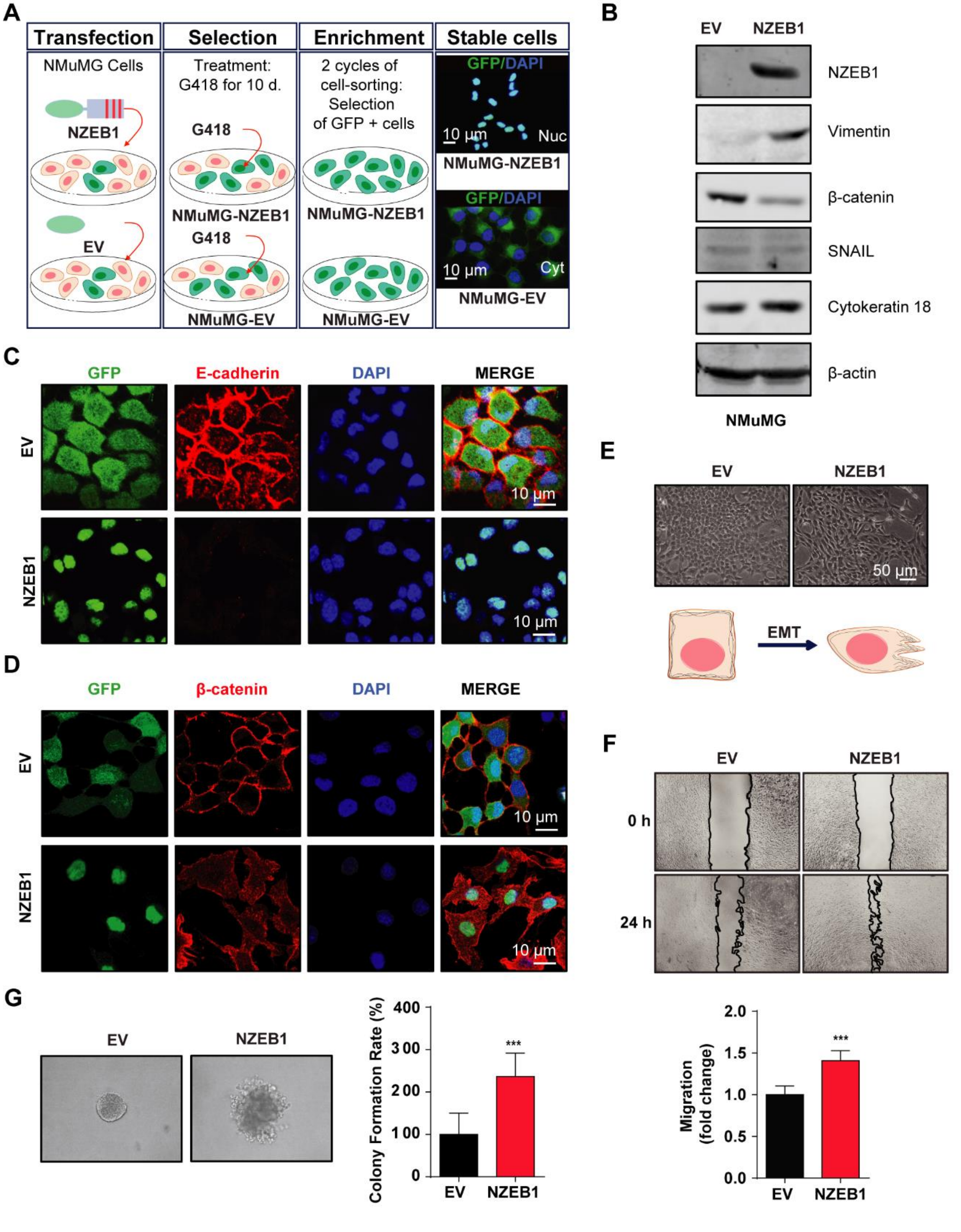

Supplementary Figure 1

SUPPLEMENTARY FIGURE 1. Stable expression of NZEB1 triggers Epithelial to Mesenchymal Transition features. (A) Scheme of the stable NMuMG cells generation expressing GFP-NZEB1 (NMuMG-NZEB1) or GFP alone (NMuMG-EV). Nuclei were stained with DAPI (blue). Nuclear (*NUC*) or Cytosolic (*CYT*) location. Magnification: 100X, scale bar: 10 µm. **(B)** Western blots for NZEB1, Vimentin, β-catenin, SNAIL and cytokeratin 18 in NMuMG epithelial cells stably expressing NZEB1 or GFP-Empty Vector (EV). Confocal microscopy analysis of E-cadherin (*red*) **(C)** or β-catenin (*red*) **(D)** subcellular localization in stable NZEB1 or EV NMuMG cell lines. Nuclei were stained with DAPI (*blue*). Magnification: 60X, scale bar: 10 µm. **(E)** Phase-contrast microscopy analysis of NMuMG NZEB1 cells. Magnification: 20X. Scale bar: 50 µm. **(F)** Migration capacity analysis by the wound healing assay in NMuMG-NZEB1 or EV NMuMG cell lines. **(G)** Soft agar colony formation assay in NMuMG-NZEB1 cells. The graph represents the colony formation rate (%) of NZEB1 NMuMG cells relativized to the control cell line (EV). Results are expressed as mean ± S.D. *ns*, not significant, (*** p≤0,001).

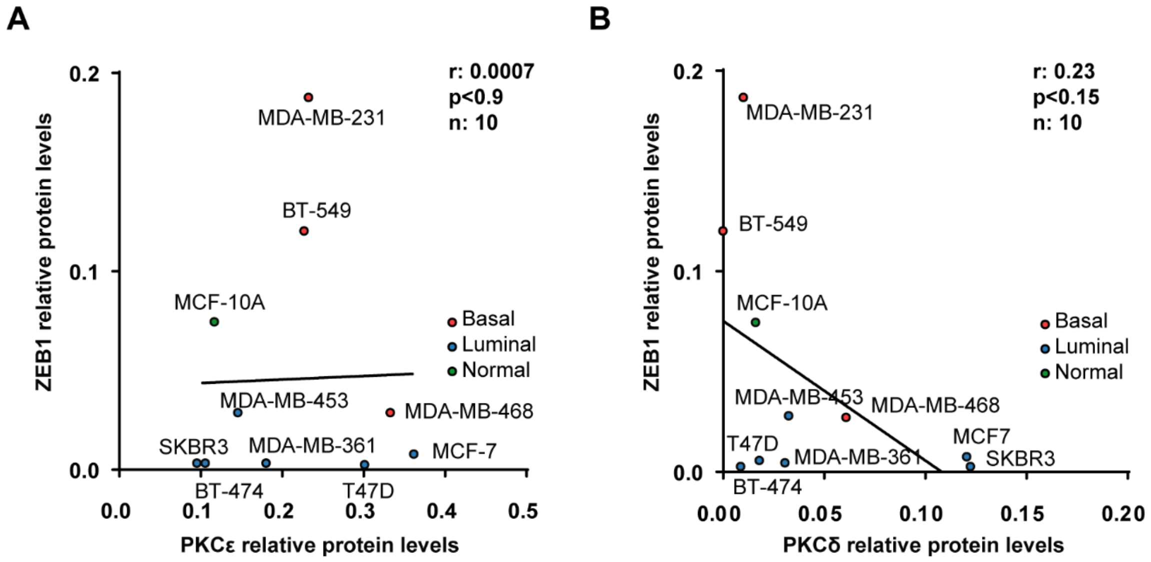

SUPPLEMENTARY FIGURE 2. (A-B) Linear regression between the protein expression levels of PKCε or PKCδ and ZEB1 in the entire set of breast cancer cell lines analyzed.

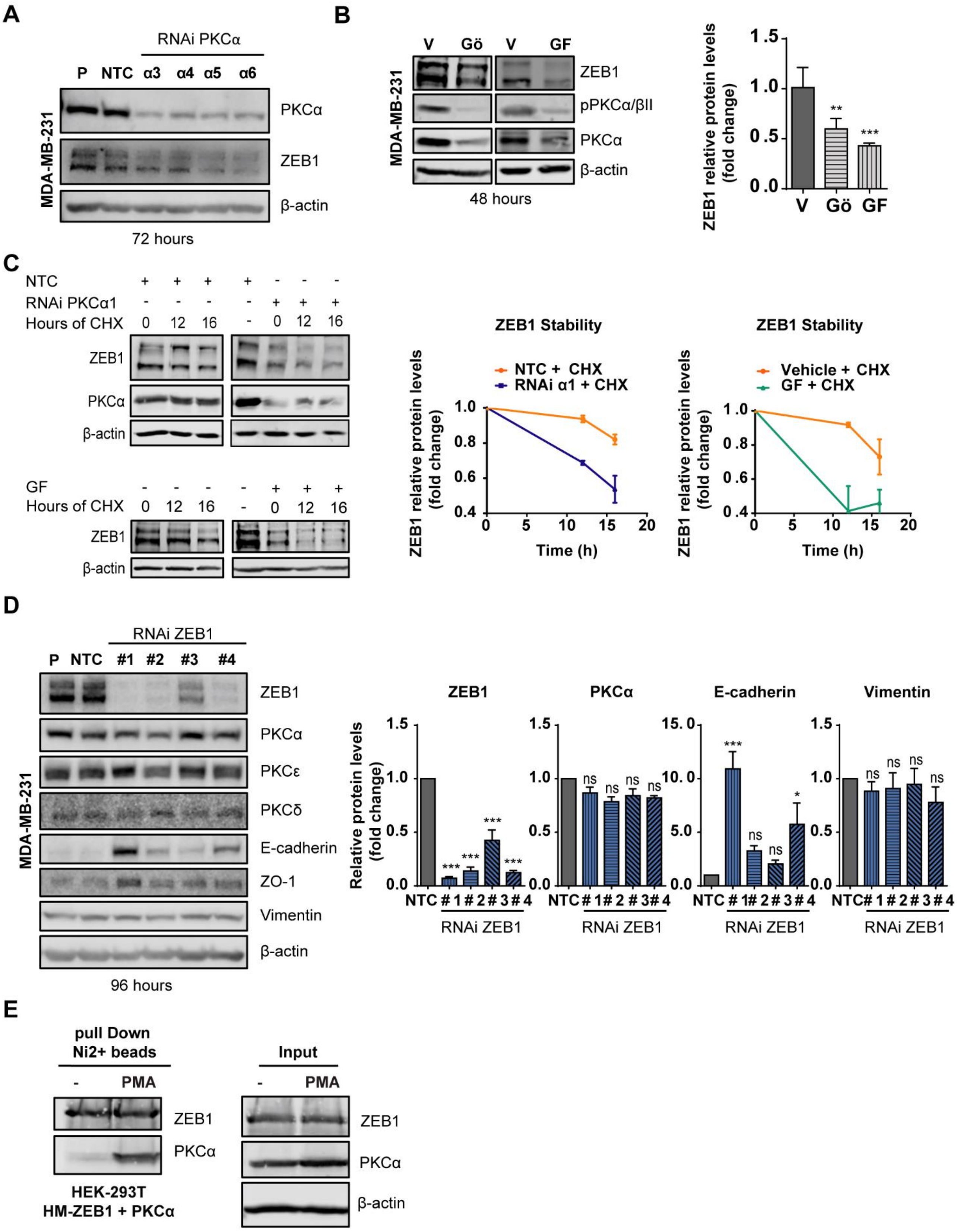
A
RNAi PKCα
P NTC α3 α4 α5 α6
PKCα
ZEB1
β-actin
MDA-MB-231
72 hours
B
V Gö V GF
ZEB1
pPKCα/βII
PKCα
β-actin
MDA-MB-231
48 hours
ZEB1 relative protein levels (fold change)
V Gö GF
C
NTC
RNAi PKCα1
Hours of CHX 0 12 16 - 0 12 16
ZEB1
PKCα
β-actin
GF
Hours of CHX 0 12 16 - 0 12 16
ZEB1
β-actin
ZEB1 Stability
NTC + CHX
RNAi α1 + CHX
ZEB1 relative protein levels (fold change)
Time (h)
ZEB1 Stability
Vehicle + CHX
GF + CHX
ZEB1 relative protein levels (fold change)
Time (h)
D
RNAi ZEB1
P NTC #1 #2 #3 #4
ZEB1
PKCα
PKCε
PKCδ
E-cadherin
ZO-1
Vimentin
β-actin
MDA-MB-231
96 hours
ZEB1
PKCα
E-cadherin
Vimentin
Relative protein levels (fold change)
NTC # 1 # 2 # 3 # 4
RNAi ZEB1
E
pull Down Ni2+ beads
- PMA
ZEB1
PKCα
HEK-293T
HM-ZEB1 + PKCα
Input
- PMA
ZEB1
PKCα
β-actin

SUPPLEMENTARY FIGURE 3. Validation of PKCα-mediated modulation of ZEB1 levels. MDA-MB-231 cells were transfected with specific PKCα RNAi duplexes (α3, α4, α5 and α6) or a non-target control (NTC) for 72 hours. Parental cell line (P) was used as internal control **(A)** or were treated with pharmacological inhibitors of pan-PKCs (*GÖ 6983* or *GF109203X) (5µM)* for 48hs **(B)** Western blot analysis for ZEB1and PKCα was carried out 72 h later. p-PKC α/βII were used as internal controls **(B)**. The graphic represent protein levels (fold change) normalized to β-actin and relativized to the DMSO treatment (vehicle: V). **(C)** Protein stability assay. MDA-MB-231 cells were transfected with specific PKCα siRNAs for 72 hours or treated with the pharmacological pan-PKCs inhibitor GF109203X (5µM) for 48 hours in combination with cycloheximide (CHX: 25ug/ml) for the last 12 or 16 hours. Western blot analysis for ZEB1and PKCα. The graphs represents the relative protein levels of ZEB1 within the time-frame of the experiment. The initial value is the maximum expression of ZEB1 when the treatment with CHX started. **(D)** MDA-MB-231 cells were transfected with specific ZEB1 siRNAs (#1, #2, #3 and #4) or NTC for 96 hours. The parental cell line (P) was used as internal control. Western blot analysis for ZEB1, PKCα, PKCε, PKCδ and the EMT markers. Blue bar graphics represent the relative protein levels of PKCα, ZEB1, E-cadherin and vimentin (fold change), normalized to β-actin and relativized to the NTC control. **(E)** Pull down with Ni2+ beads of exogenous ZEB1 in HEK-293T cells co-transfected with HM-ZEB1 and PKCα. Previously the cells were treated with the PKC activator (PMA) for 15 minutes. Western blots were performed using a specific anti PKCα and anti ZEB1 antibodies. Results are expressed as mean ± S.D. *ns*, not significant; * $P \leq 0.05$; ** $p \leq 0.01$; *** $p \leq 0.001$.

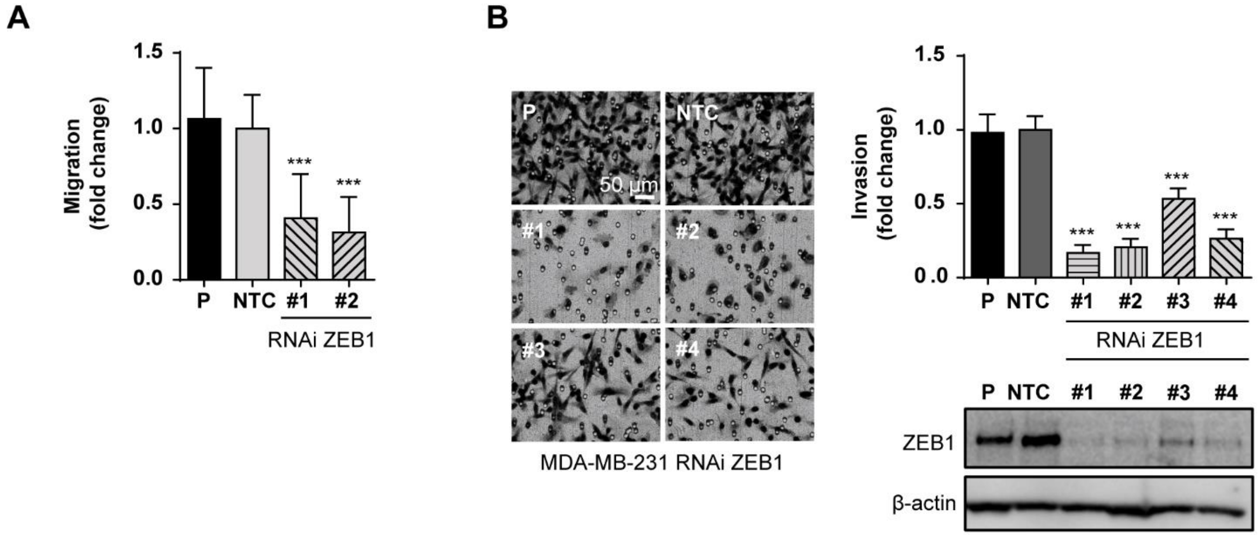

SUPPLEMENTARY FIGURE 4. Complementary control experiments showing the impaired migratory and invasion capacity of ZEB1 downregulated cells. **(A)** MDA-MB-231 cells were transfected with specific ZEB1 siRNAs (#1 and #2) or a non-target control (NTC). Migration capacity analysis by wound healing assay. The graphic represent the migrated area relativized to the control (NTC). **(B)** MDA-MB-231 cells were transfected with four specific ZEB1 siRNAs (#1, #2, #3 and #4) a NTC for 72 hours. Matrigel invasion assay. The graphic represent invading cells per field relativized to the control (NTC). Results are expressed as mean ± S.D. *** $p \leq 0.001$.
